# Evidence towards Improved Estimation of Respiratory Muscle Effort from Diaphragm Mechanomyographic Signals with Cardiac Vibration Interference Using Sample Entropy with Fixed Tolerance Values

**DOI:** 10.1371/journal.pone.0088902

**Published:** 2014-02-19

**Authors:** Leonardo Sarlabous, Abel Torres, José A. Fiz, Raimon Jané

**Affiliations:** 1 Institut de Bioenginyeria de Catalunya (IBEC), Barcelona, Spain; 2 CIBER de Bioingeniería, Biomateriales y Nanomedicina (CIBER-BBN), Barcelona, Spain; 3 Department ESAII, Universitat Politècnica de Catalunya, Barcelona, Spain; 4 Department of Pneumology, Germans Trias i Pujol Hospital, CIBERES, Badalona, Spain; University of Adelaide, Australia

## Abstract

The analysis of amplitude parameters of the diaphragm mechanomyographic (MMGdi) signal is a non-invasive technique to assess respiratory muscle effort and to detect and quantify the severity of respiratory muscle weakness. The amplitude of the MMGdi signal is usually evaluated using the average rectified value or the root mean square of the signal. However, these estimations are greatly affected by the presence of cardiac vibration or mechanocardiographic (MCG) noise. In this study, we present a method for improving the estimation of the respiratory muscle effort from MMGdi signals that is robust to the presence of MCG. This method is based on the calculation of the sample entropy using fixed tolerance values (fSampEn), that is, with tolerance values that are not normalized by the local standard deviation of the window analyzed. The behavior of the fSampEn parameter was tested in synthesized mechanomyographic signals, with different ratios between the amplitude of the MCG and clean mechanomyographic components. As an example of application of this technique, the use of fSampEn was explored also in recorded MMGdi signals, with different inspiratory loads. The results with both synthetic and recorded signals indicate that the entropy parameter is less affected by the MCG noise, especially at low signal-to-noise ratios. Therefore, we believe that the proposed fSampEn parameter could improve estimates of respiratory muscle effort from MMGdi signals with the presence of MCG interference.

## Introduction

Mechanomyographic (MMG) signals are used to record and evaluate the mechanical activity of the skeletal muscles during contraction. These signals, represent a non-invasive technique for measuring the low-frequency lateral oscillations of muscle fibers during contraction. Furthermore, it has been found that in striated muscle there is a positive correlation between amplitude parameters of the MMG signal and the force produced by the muscle [1], [2], [3], [4].

Like other skeletal muscles, the diaphragm vibrates laterally during contraction. These muscle vibrations can be recorded using microphones, piezoelectric sensors or accelerometers placed over the lower chest wall in the zone of apposition of the diaphragm to the rib cage [5]: the diaphragm MMG (MMGdi) signal. The main frequency content of this signal lies between 5 and 25 Hz [6], [7]. During the recording of MMGdi signals several potential sources of contamination in addition to environmental noise must be eliminated or controlled, cardiac vibrations, detected in seismocardiograms or mechanocardiograms (MCGs), typically causing the most interference. MCGs have a deterministic and repetitive pattern, and contain clearly defined points associated with the cardiac cycle [8], [9], [10]. The MCG signal can be detected in both hemidiaphragms, being stronger on the left side [11], and its frequency content is below 20 Hz [12], [13]. Therefore, there is an overlap between the frequency content of the MMGdi and MCG signals, and hence the potential for interference.

Clinically, the measure of respiratory muscle strength is valuable to detect muscle weakness and to quantify its severity. The strength of these muscles is commonly assessed by measuring maximal inspiratory mouth pressure (IP), but values obtained in this way could be underestimated [14]. Analysis of MMGdi amplitude is a useful alternative approach for assessing respiratory muscle strength [6], [7].

Sample entropy (SampEn), developed by Richman and Moorman [15], is widely used to estimate complexity and regularity in biomedical signals, having been found to be useful for the analysis of this type of signal in many fields [15] [16], [17], [18], [19], [20]. SampEn is an improved measure of regularity to overcome the inherent bias observed in approximate entropy [21] because of the self-matching of vectors. Specifically, SampEn does not count self-matches and, thereby, removes the bias and is more robust to noisy and short data series than approximate entropy.

The amplitude of the MMGdi signal is usually estimated by the average rectified value (ARV) or the root mean square (RMS). These amplitude estimators are, however, affected by various types of noise such as: motion artifacts due to breathing, impulsive noise, spurious spikes, and MCG interference, among others. In [6], [7], and [22], it was observed that traditional complexity parameters calculated using a fixed quantization interval and over a moving window are more closely related to amplitude variations than to complexity variations of the signal. In particular, the multistate Lempel-Ziv index [6] and approximate entropy [7] of MMGdi signals provided a better measure of respiratory effort (i.e., respiratory muscle strength) than the traditional amplitude parameters as the ARV and RMS. On the other hand, it was observed that multistate Lempel-Ziv index was less affected by impulsive noise [6] and SampEn was less affected by spurious spikes [22].

The objective of this study was to overcome the influence of MCG interference to obtain an accurate amplitude estimation of MMGdi signals applying the SampEn method over a moving window and with fixed tolerance values (fSampEn). These tolerance values are in the range of 0.1–1 times the global standard deviation of the original signal, and they do not depend on the standard deviation of each moving window used for the calculation. In this paper, we describe the behavior of fSampEn with simulated MMGdi signals with different signal-to-noise ratio (SNR) distributions. Furthermore, we apply also this technique to recorded MMGdi signals with different inspiratory loads. We also assess the feasibility of distinguishing respiratory cycles using fSampEn method compared to the ARV and RMS parameters. Finally, we evaluate the robustness of these amplitude estimators in presence of MCG interference and its relationship with respiratory muscle strength.

## Methods

### Sample entropy

SampEn is a measure that depends on the conditional probability of two sequences that are similar for *m* samples (where *m* is a positive integer) remaining similar within a tolerance *r* in the next sample *m*+1. A data sequence with many repetitive patterns (i.e., that is predictable or relatively regular) has a small value of SampEn, while one with few repetitive patterns (i.e., that is less predictable or more irregular) has a larger value of SampEn. Given a signal *x(n) = x(1), x(2),…, x(N)* of length *N*, and defined *r* and *m*, SampEn (*m*, *r*, *N*) is calculated as follows [15]:

1. Form the *m*-vector sequences X_m_(1)…X_m_(*N-m+1*), which can be defined by X_m_(*i*) = [*x(i)*, *x(i+1)*,…, *x(i+m-1)*]; where 1≤*i*≤*N-m+1*. These vectors represent *m* consecutive values of *x(n)*.
2. Define the distance between X_m_(i) and X_m_(j) as the maximum absolute difference between their respective scalar components:
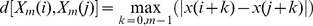
(1)
3. Define *B_i_* for each X_m_(i) as the number of *j*(1≤*j*≤*N-m, j*≠*i*) such that 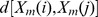
≤*r*, and then define:
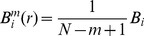
(2)

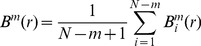
(3)
4. Increase the dimension to *m*+1, and define 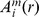 and 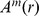 for each X_m+1_(i) such that 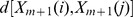
≤*r*:
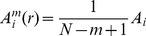
(4)

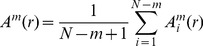
(5)
5. Then, estimate SampEn as:
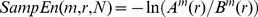
(6)

### Sample entropy with fixed *r* values

In this paper, we propose the calculation of SampEn over a moving window and using fixed *r* values and *m* = 1 (fSampEn). These *r* values are in the range of 0.1 to 1 times the global standard deviation of the original signal, and they do not depend on the standard deviation of each moving window used for the calculation. Once the fixed *r* has been determined, the fSampEn is calculated following the steps and the equations described above.

### Synthesized diaphragm MMG signal

The MMG signals are composed of low-amplitude vibrations generated during muscular contraction. These low-amplitude vibrations are related to the mechanical activity of the muscle [3]. The MMG amplitude progressively increases with contraction effort [3], [23], although this increase is not monotonous and it is muscle dependent. The frequency content of the MMG signal is mainly in the range between 5 to 50 Hz. In the case of the MMGdi signal, the frequency content lies mainly between 5 and 25 Hz and the amplitude varies cyclically with a frequency determined by the respiratory rate [6].

To better understand how fSampEn detects amplitude variations, we generated a synthesized signal based on experimental MMGdi data. The synthesized signal describes similar characteristics to those of the MMGdi signals acquired during an incremental inspiratory load respiratory test. To the authors' knowledge, no published models describe the properties of the MMG signal during voluntary contractions. Other researchers have developed models to simulate the behavior of the MMG signal generated during single motor unit contractions [24], [25], and in contractions evoked by artificial muscle stimulation (during artificial stimulation several motor units being activated simultaneously and behaving as a single large motor unit) [27], [28], [29]. However, the behavior of the MMG signal in such contractions is completely different from that during voluntary contractions: the simultaneous contraction of the motor units makes the waveform of the artificially evoked MMG signal more deterministic than random [27], [28].

**Figure 1:**
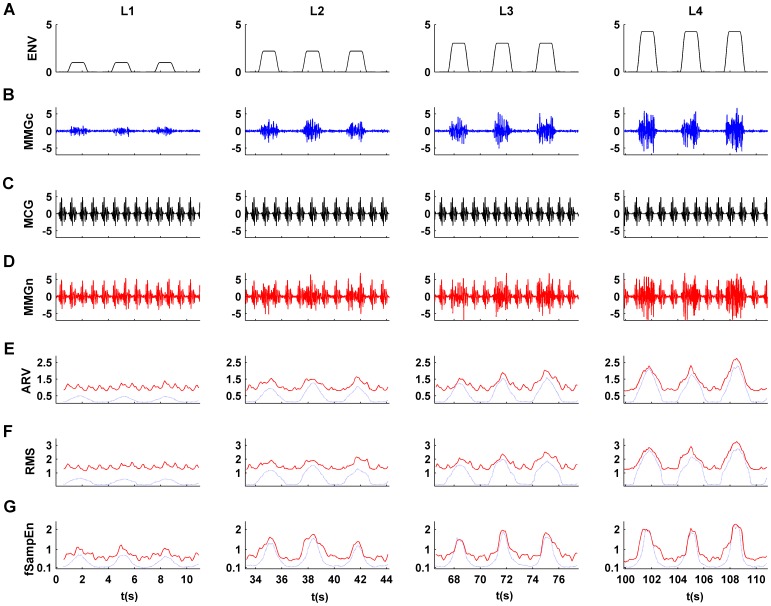
Synthetic signal with different levels of inspiratory load. (A) Envelope signal (ENV), (B) synthesized MMGdi signal clean of cardiac noise (MMGc), (C) synthesized cardiac vibration signal (MCG), (D) synthesized MMGdi signal with cardiac noise (MMGn), (E) ARV, (F) RMS and (G) fSampEn(1,0.3,200) determined in both MMGc (blue line) and MMGn (red line) signals using a 1 s moving window.

Since most of the frequency content of MMGdi signal lies between 5 and 25 Hz and the MMG signal is random in nature [3], we assumed white Gaussian noise filtered using a zero-phase fourth-order Butterworth filter with a bandpass from 5 to 25 Hz to simulate the vibratory behavior. In order to simulate the cyclical behavior of the MMGdi signal, we first generated an amplitude modulation envelope (Figure 1A). This envelope signal (ENV) was designed to simulate the IP increments produced when the inspiratory load increases. Specifically, the ENV amplitude increments were equivalent to those produced in the MMGdi signals for the four incremental inspiratory loads studied. Each inspiratory load consisted of 10 simulated respiratory cycles of the same duration (3.33 s approximately). The respiratory rate and total duration of the ENV signal were 18 cycles per minute and 133.33 s, respectively. The simulated inspiratory periods comprise 50% of the total respiratory period. The selected respiratory rate and inspiratory periods where selected based on data from a study of breathing patterns in healthy subjects [30]. During inspiration, the amplitude of the MMGdi signal progressively increases until reaching a plateau and then gradually decreases to the rest level. To simulate this behavior, each simulated inspiratory period was divided into three phases: (1) rise (25%), (2) plateau (50%) and fall (25%). The rising and falling phases were simulated by means of half-Hanning windows. Then, multiplying the simulated random MMGdi signal with constant amplitude by ENV, we obtained a modulated amplitude signal whose respiratory rate was similar to the MMGdi signals. Finally, to simulate the non-cardiac biological noise present in the MMGdi signal at rest we added background white Gaussian noise filtered through a zero-phase fourth-order Butterworth filter with a bandpass from 5 to 50 Hz to obtain the synthesized MMGdi signal (Figure 1B) clean of cardiac noise (MMGc). The amplitude of this background noise was equivalent to the amplitude of the MMGdi signal recorded during apnea in the portion of signal where no heart activity is present.

To generate the synthesized cardiac vibration signal (MCG), we simultaneously recorded the electrocardiographic and MMGdi signals during apnea in a healthy subject. During apnea the respiratory muscle activity is minimal so that this MMGdi signal mainly contains MCG activity. The MCG has a stable and repetitive pattern and contains clearly defined points associated with the cardiac cycle [8], [9],[10]. To obtain a good estimation of the MCG pattern we generated an MCG signal using a template. Specifically, we obtained this template by averaging 70 cardiac cycles extracted from the MMGdi signal, using the position of R-peaks in the electrocardiographic signal to align the cycles. Next, we generated an impulse train synchronized with these R-peak positions. Finally, we obtained the synthesized MCG signal by the convolution of the MCG template and the impulse train (Figure 1C).

The complete synthesized signal with noise (MMGn) was generated by adding the MMGc and MCG signals (Figure 1D). For each simulated respiratory load, we considered a different SNR: L1(−8.7 dB), L2(−1.7 dB), L3(0.6 dB) and L4(3.8 dB). The sampling frequency used to generate all the signals was 200 Hz.

### Recorded biomedical signals

IP signal and MMGdi signal were simultaneously recorded while increasing the inspiratory load. These measurements were taken in a healthy subject with his written consent, and with the approval of the Ethics Committee of Hospital del Mar, Barcelona, Spain. The subject was required to sit quietly and breathe through a mouthpiece and a tube, while wearing a nose clip. During exhalation the tube allowed the air out with no obstruction, but during inspiration the airflow was restricted by a valve that allowed the application of different inspiratory loads. Increasing the load meant that breathing required greater respiratory muscle effort, and hence, triggered an increase in the intensity of the MMGdi component of the signal. Moderate to high inspiratory loads were used to obtain different SNR ratios: 100, 150, 200 and 250 g. A physician instructed the subject to perform the protocol correctly, guiding him to breathe at a constant rate and depth.

The IP signal was recorded using a pressure transducer (Digima Premo 355, Special Instruments, Germany) placed in the tube through which the subject breathed. The MMGdi signal was recorded using a capacitive accelerometer (8312B2, Kistler, Switzerland) placed on the chest surface, between the seventh and eighth intercostal spaces in the right anterior axillary line. Signals were amplified, analog filtered, digitized with an A/D system of 12 bits at a sampling frequency of 2 kHz and decimated at a sampling rate of 200 Hz.

**Figure 2:**
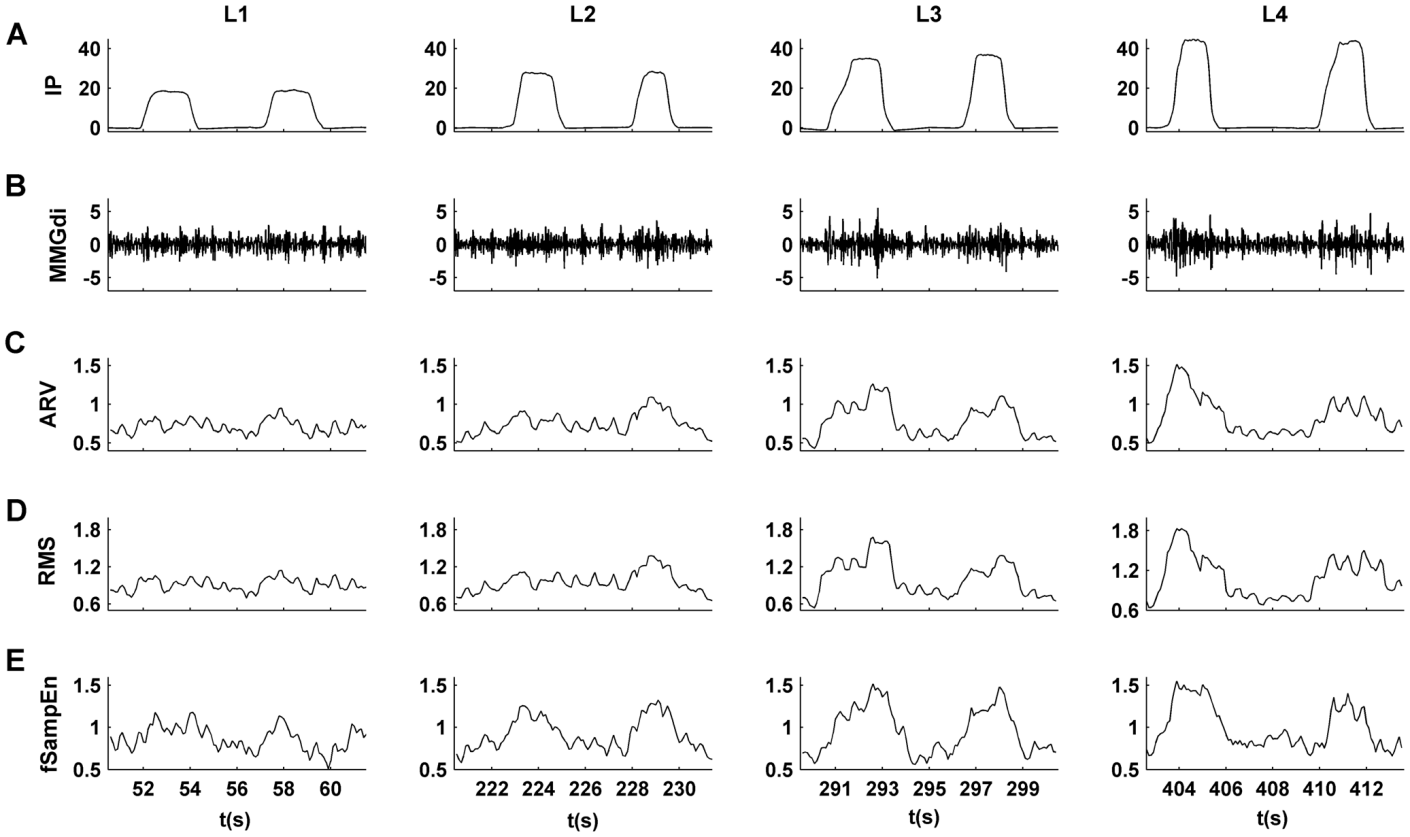
Recorded signal with different levels of inspiratory load. (A) Inspiratory mouth pressure (IP) signal, (B) filtered MMGdi signal respiratory (5–25 Hz) recorded during an incremental inspiratory load protocol. The inspiratory load increases from 100 to 250 g. (C) ARV, (D) RMS and (E) fSampEn(1,0.3,200) determined in MMGdi signal using a 1 s moving window.

Figures 2A and B show the IP signal and filtered recorded diaphragm MMG signal (MMGdi). The MMGdi signal was filtered through a zero-phase fourth-order Butterworth filter with a bandpass from 5 to 25 Hz. The duration of the signal was 485 s covering four inspiratory loads: 100 (126 s), 150 (122 s), 200 (115 s) and 250 (122 s) g. Each load was placed for approximately 21 respiratory cycles.

### Methods for evaluation of the fSampEn parameter

To evaluate the behavior of fSampEn as an MMGdi signal amplitude estimator and the effect of cardiac noise on this amplitude estimation, we used the Pearson's correlation coefficient (R) and the mean relative error (MRE).

First, for the synthesized MMGdi signal, we calculated the R between the ENV signal and the ARV, RMS and fSampEn parameters over the MMGn signal. In the case of fSampEn, the R values were investigated as a function of the tolerance value *r*. These R values are calculated separately for the four SNRs analyzed (i.e., for the four simulated loads) and reflect the capability of the methods to detect the amplitude variations produced by cyclical nature of breathing for different SNRs (not considering the amplitude variations due to the load increase).

In addition, the MRE between the synthesized MMGc and MMGn signals was calculated for every inspiratory cycle in the three amplitude parameters under investigation (ARV, RMS and fSampEn). For an inspiratory cycle *i* of length *N*, where *Xc(n)* and *Xn(n)* for *n = 1,…,N* are the amplitude estimations of clean and noisy signals, respectively, the MRE is given by:
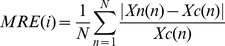
(7)The average and standard deviation of this error, estimated for every inspiratory cycle, was calculated separately for the inspiratory cycles of the four simulated loads, and for different values of *r* in the case of fSampEn.

In the case of the recorded signals, similar to the analysis of the synthesized signals, we calculated the R between the IP signal and the three parameters under investigation over the MMGdi signal for the four inspiratory loads. The R for fSampEn was calculated as a function of *r*. Unlike for the synthesized signals, however, it is not possible to compute the MRE since we do not have the clean MMGdi signal (that is, without MCG activity).

Finally, to evaluate the relationship between the respiratory muscle force and the amplitude of the recorded MMGdi signal, the R between the IP signal and the three parameters under investigation calculated over the MMGdi signal was recalculated considering the whole signal (without dividing it into portions corresponding to different loads). In this case, the R mainly reflects the relationship between the parameters analyzed and amplitude variations due to changing the inspiratory load (although it is also influenced by the amplitude variations produced by the breathing cycle).

## Results

### Fixed sample entropy as an amplitude estimator

In Figures 1 and 2, we show an example of the waveforms of the ARV (Figures 1E and 2C), RMS (Figures 1F and 2D) and fSampEn (Figures 1G and 2E) obtained from the synthesized (MMGc and MMGn) and recorded MMGdi signals, respectively. The waveforms were obtained using a 1 s moving window with an overlap of 90%. The values of fSampEn were calculated using a tolerance value of 0.3 times the standard deviation of the entire signal. In this case, we observed that the amplitude variation due to the respiratory cycles was best defined with fSampEn. That is, the entropy parameter provides a better amplitude estimation than the ARV or RMS parameters, especially for low SNR.

### Effect of cardiac noise in the synthesized MMG signals

**Figure 3:**
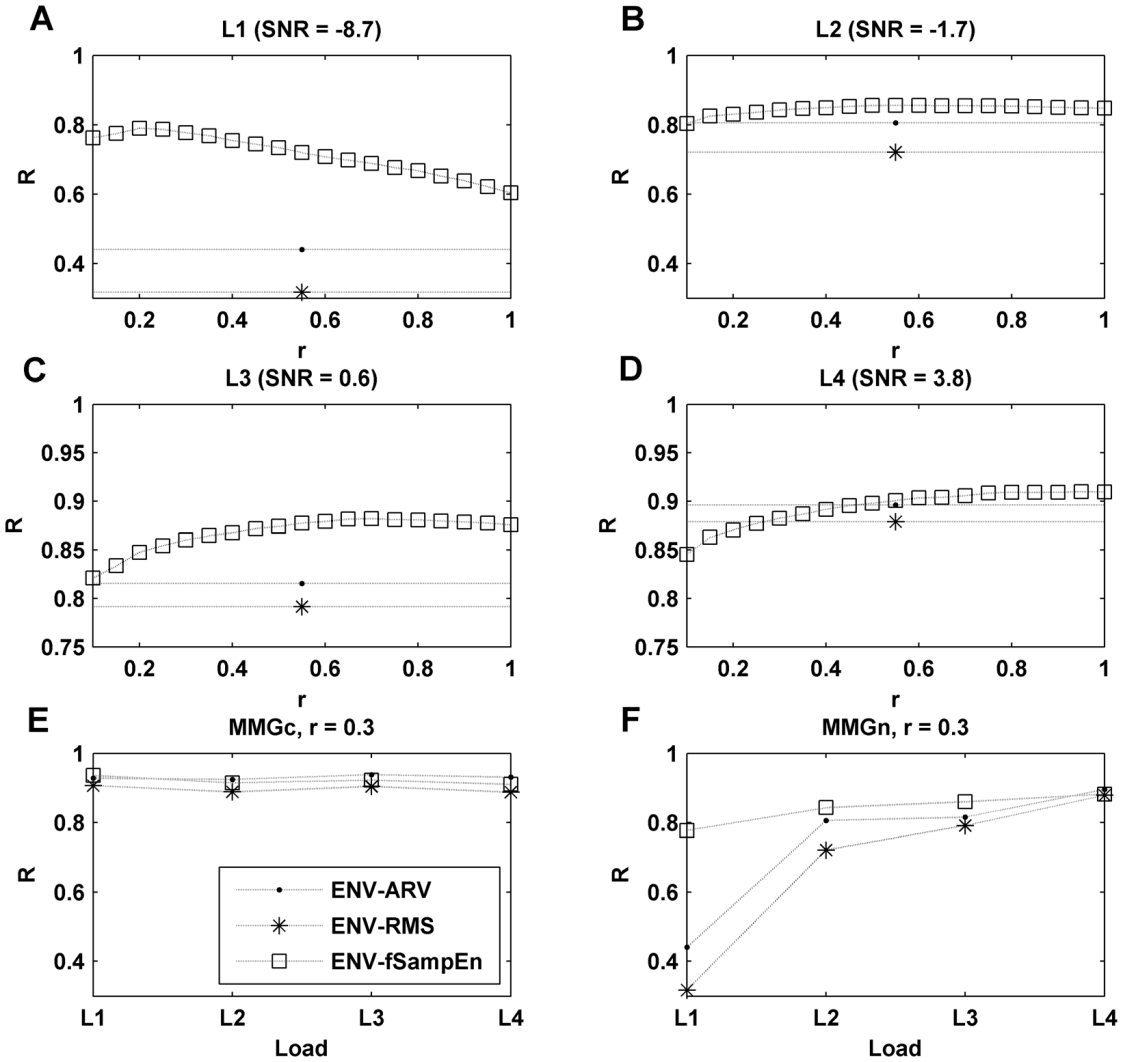
Correlation between ENV and MMGn signals. Pearson's correlation coefficient (R) between ENV signal and the amplitude parameters (ARV, RMS and fSampEn) estimated in MMGn signal as a function of simulated inspiratory load. fSampEn was determined for *r* values between 0.1–1 (plots A to D), and for *r* = 0.3 in MMGc (E) and MMGn (F) signals. Each simulated respiratory load had a different SNR distribution: L1(−8.7 dB), L2(−1.7 dB), L3(0.6 dB) and L4(3.8 dB).

Changes in R between the ENV signal and the ARV, RMS and fSampEn parameters calculated over the MMGn signal are shown in Figure 3. The R values are shown for all SNRs analyzed and as a function of *r* for fSampEn. Values of *r* analyzed were in the range of 0.1 to 1 times the global standard deviation of the entire signal. For low SNR (Figure 3A), we observe that R is higher for fSampEn than for either the ARV or RMS parameters. This means that the entropy parameter performs better for determining the presence of respiratory cycles (see load L1 in Figure 1). For high SNRs (Figure 3D), R is high for all amplitude estimators, and slightly higher for fSampEn for values of *r* greater than 0.5.

Figures 3E and F show the R between the ENV signal and the ARV, RMS and fSampEn parameters calculated over the synthesized MMGc and MMGn signals, respectively. The R values for fSampEn were calculated using *r* = 0.3. The values showed for MMGn signals (Figure 3F) are the values shown in Figures 3A-D for *r* = 0.3. As can be observed, when no MCG noise is present (Figure 3E) the R values are very high, regardless of the load. However, when the MCG noise is present (Figure 3F), the R values fall rapidly as the SNR decreases. This decrease is more pronounced for the ARV and RMS parameters than for fSampEn.

**Figure 4:**
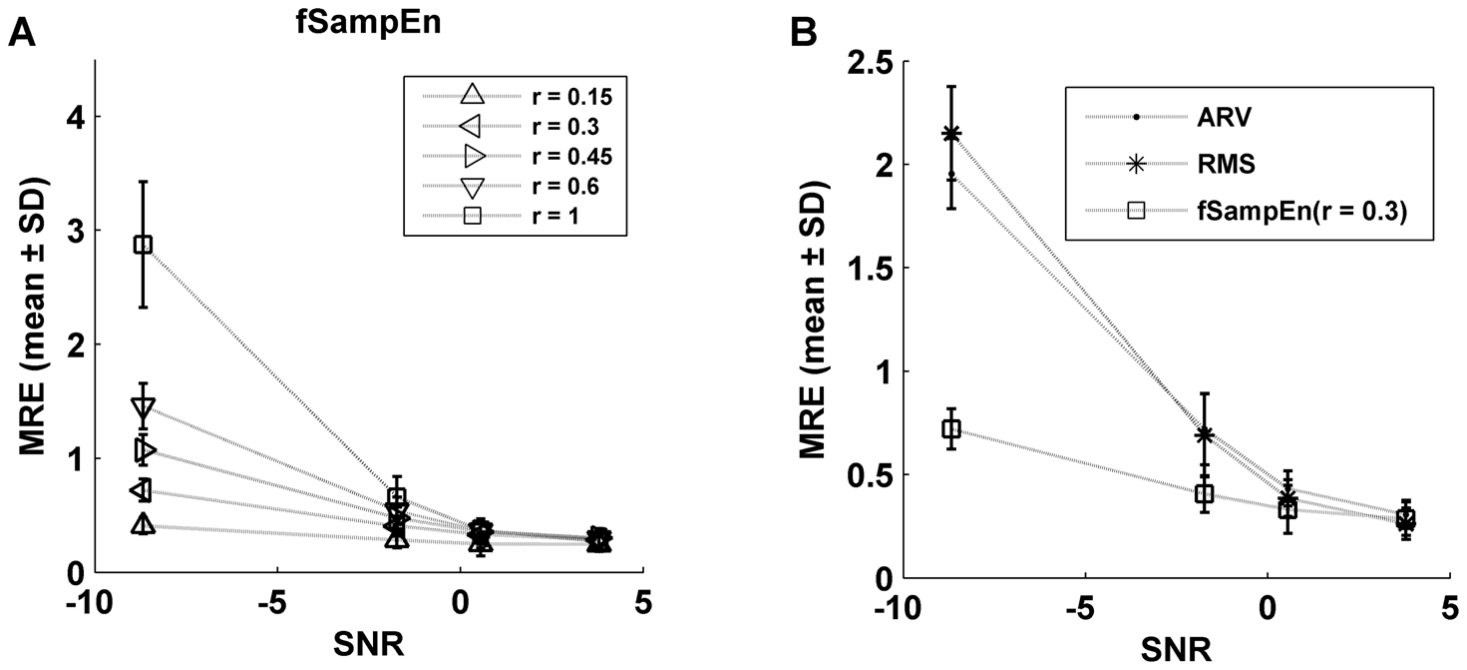
Mean relative error between MMGc and MMGn signals. (A) Average and standard deviation of the mean relative error (MRE) between the synthesized MMGc and MMGn signals for the fSampEn calculated for values of *r* = 0.15, 0.3, 0.45, 0.6 and 1. (B) MRE between the synthesized MMGc and MMGn signals for ARV, RMS and fSampEnwith *r* = 0.3.

In Figure 4 we show the average and standard deviation of MRE between the synthesized MMGc and MMGn signals in the three parameters under investigation calculated separately for the four simulated loads. The MRE obtained for fSampEn for values of *r* = 0.15, 0.3, 0.45, 0.6 and 1 is shown in Figure 4A. It can be seen that increasing *r*, the mean value of the MRE also increases for fSampEn. In Figure 4B, we compare the means and standard deviations of the MRE of the ARV and RMS parameters, with those of the fSampEn calculated with a tolerance value *r* = 0.3. We observe that the average value of MRE is considerably smaller in the entropy parameter, in particular at low SNRs.

### Effect of cardiac noise in the recorded signals

**Figure 5:**
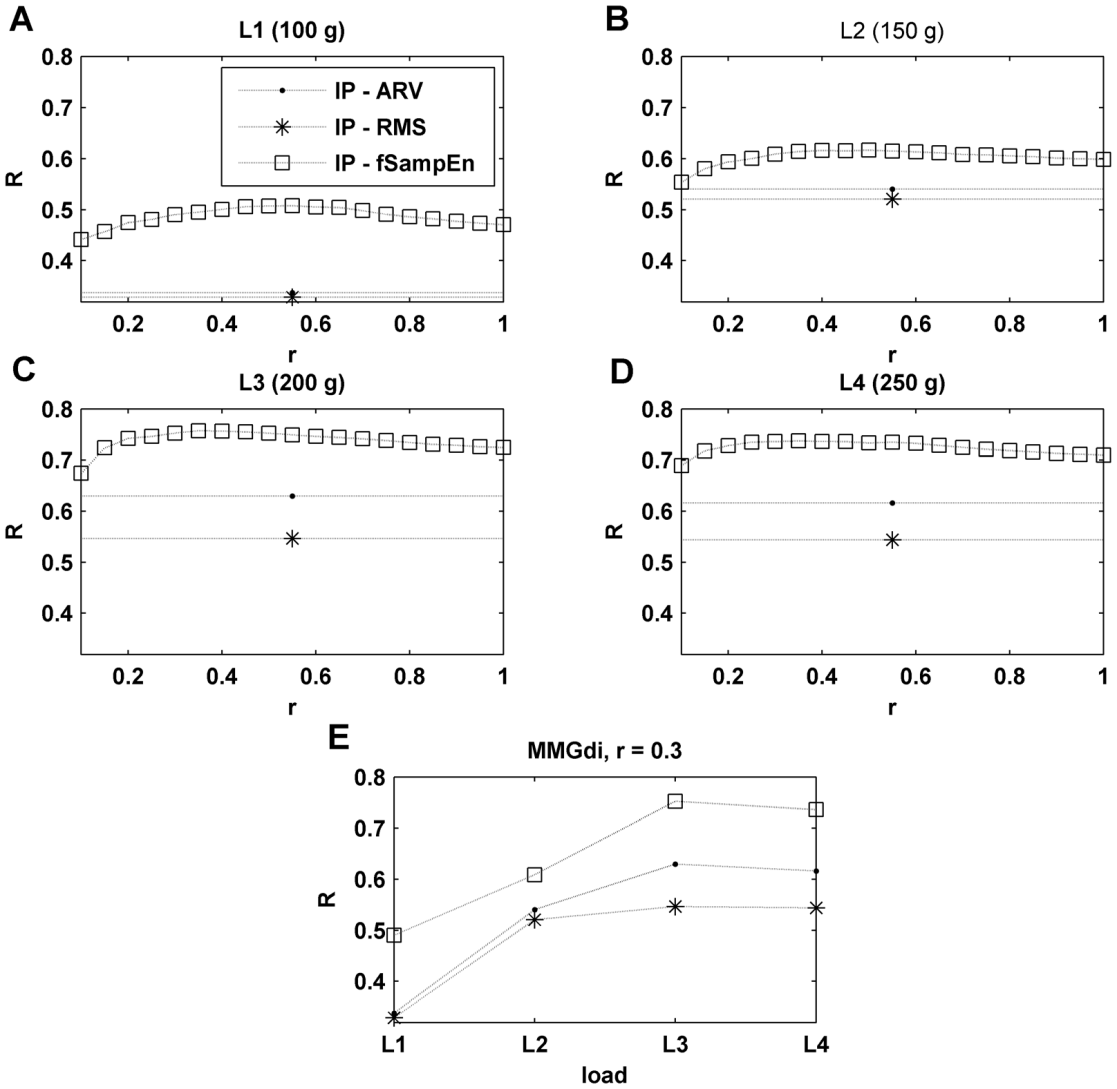
Correlation between IP and MMGdi signals. Pearson's correlation coefficient (R) between the IP signal and the amplitude parameters (ARV, RMS and fSampEn) estimated in MMGdi signal as a function of inspiratory load. fSampEn was determined for *r* values between 0.1–1 (plots A to D), and for *r* = 0.3 (plot E).

Figure 5 shows the change in R between the IP signal and the three parameters under investigation over the MMGdi signal, for the four inspiratory loads. The R for fSampEn is shown as a function of *r*. Similar to the behavior observed with the synthesized signals, for a low load (Figure 5A) we observe a stronger correlation for the entropy parameter than for the ARV and RMS. In this case, this trend is also observed for a high load (Figure 5D) for almost all the tolerance values analyzed.

Figure 5E shows the R values presented in Figures 5A–D, with the fSampEn focused on *r = 0.3*. In this case, the R values are shown as a function of inspiratory load. As can be observed, the correlation values are smaller at low loads (low SNR), but in this case, unlike in the synthesized signals, the correlation values were significantly higher for the entropy parameter for all loads (even at high loads).

### Evaluation of respiratory muscle force

**Figure 6:**
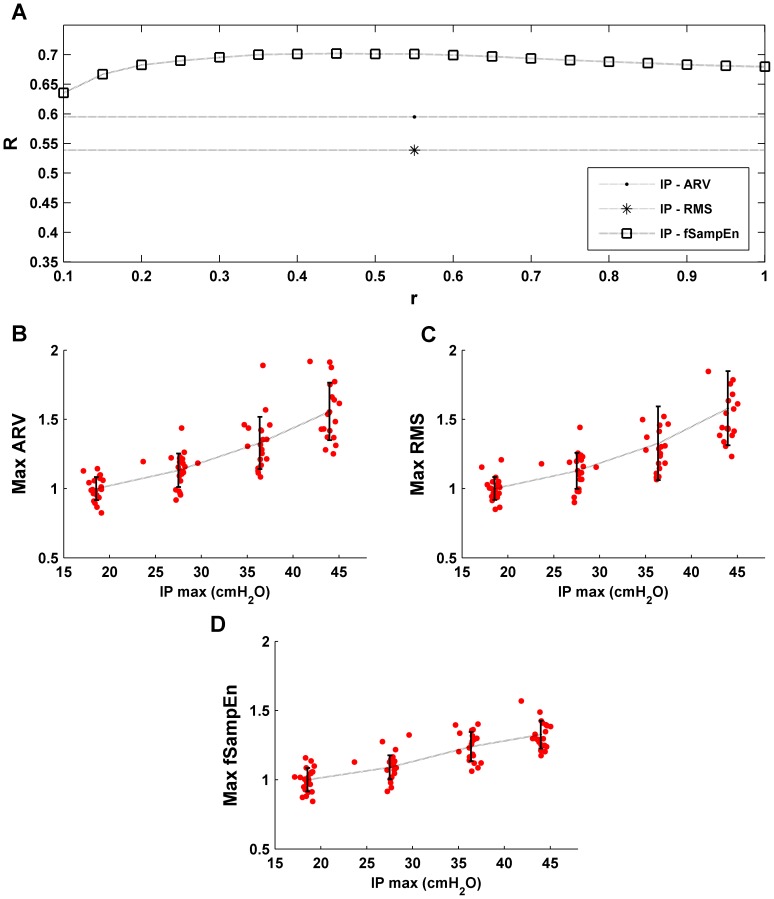
Evaluation of respiratory muscle force. (A) Evaluation of the respiratory muscle force by means of the Pearson's correlation coefficient (R) between the IP signal and the amplitude parameters (ARV, RMS and fSampEn) estimated considering the whole MMGdi signal. fSampEn was determined for *r* values between 0.1–1. Scatter plots of the maximum values of the IP signal and the maximum values of the amplitude parameters: (B) ARV, (C) RMS, (D) fSampEn determined using *r* = 0.3, as a function of respiratory load.

To evaluate the relationship between the respiratory muscle force and the amplitude of the recorded MMGdi signal, we investigated the R between the IP signal and the three parameters under investigation over the MMGdi signal this time considering the whole signal (without dividing it into the portions corresponding to different loads). Figure 6A shows the evolution of the R between the IP signal and all parameters analyzed calculated over the MMGdi signal. As before, the correlation for fSampEn is shown as a function of *r*. As we can observe, fSampEn is more strongly correlated with the IP signal than the ARV and RMS parameters. The maximum R values were obtained for *r* values between 0.3 and 0.6.

Figures 6B, C and D are scatter plots of the maximum values of IP signal and all parameters analyzed (ARV, RMS and fSampEn) as a function of respiratory load, respectively. Values of fSampEn were calculated using *r* = 0.3. It is observed that the fSampEn behaves more linearly and has a smaller standard deviation than the ARV and RMS parameters.

## Discussion

The analysis of amplitude parameters of the MMGdi signal is a non-invasive technique to assess respiratory muscle effort [31]. The amplitude content of the MMGdi signal is usually estimated using the RMS or the ARV of the signal. Nevertheless, as corroborated in this simulation study, these estimations are greatly affected by the presence of cardiac vibration interference that overlaps in frequency with the MMGdi signal. Furthermore, an increase in respiratory muscle effort results in an increase in the intensity of the MMGdi component: that is, the SNR is variable and increases with respiratory effort.

Various methods can be applied to minimize the effect of heart vibrations in the analysis of MMGdi signals. The simplest method would be, similar to a method used in the diaphragm electromyographic signal [32], the detection and removal of the parts of the MMGdi signal with cardiac interference [33]. However, this method splits the signal and excludes portions of the signal that may contain essential information about the contractile activity of the diaphragm muscle. Adaptive noise cancelling algorithms have been also applied to reduce cardiac interference in MMGdi signals, but the operation of the adaptive canceller is based on an approximate estimation of a cardiac vibration reference signal and its performance varies considerably depending on the SNR of the signal [33], [34].

In this study, we present a method for improving the estimation of respiratory muscle effort from MMGdi signals that is robust against cardiac vibration interference. This method is based on the computation of the SampEn using fixed tolerance values (fSampEn), not dependent on the standard deviation of each moving window. In this way, the entropy measures are related to the quantity of information present in the signal: the entropy is higher if the signal covers a wide range of amplitudes or if it is highly complex. With signals where the standard deviation of the signal is not constant, the SampEn also increase with an increase of amplitude. Thus, the SampEn is not just measuring the complexity of the signal but also changes in signal amplitude. Since heart sounds have a deterministic and repetitive pattern [8],[9],[10] and MMGdi vibrations are random in nature [3], the fSampEn is less influenced by cardiac vibrations than the ARV and RMS parameters. Analysis of synthetic MMGdi signals has allowed us to explore the relationship between the amplitude of heart vibrations and the amplitude of the MMGdi signal. For low SNRs, the fSampEn shows considerably better behavior than the ARV and RMS parameters, and it also shows better behavior when small values of tolerance are used. For high SNRs, the fSampEn shows better behavior for large values of tolerance. However, we observed that increasing the tolerance value produces higher MRE between the values of fSampEn calculated over the synthesized MMG signal with and without MCG noise. This increase is more pronounced at low load (low SNRs). As the r value increases the fSampEn is less sensitive to the small changes in amplitude that are produced at low load. This behavior is in agreement with what is shown in Figure 3a, where it can be observed that the R between the entropy parameter and the ENV signal decreases with increasing *r* (unlike what occurs with high SNRs). Thus, there is a compromise in the selection of the tolerance value. It was found that a tolerance value of *r* = 0.3 was suitable in the current study for both low and high SNRs.

As an example, the fSampEn method was also applied to recorded MMGdi signals, obtaining a similar pattern of results to those with synthetic ones. Further, in this case the performance of the fSampEn is much better than that of the RMS and ARV for all the respiratory loads analyzed (for both low and high SNR). For almost all the tolerance values analyzed, the R values between the IP signal and the fSampEn were notably greater than the R values between the IP signal and the ARV and RMS parameters, indicating that this entropy parameter is a better tool to assess respiratory effort. Furthermore, in general, the variance of fSampEn is lower than the variance of the ARV and RMS parameters. These results are in agreement with a previous study comparing the approximate entropy using fixed tolerance values and the RMS of MMGdi signals acquired in an animal model (dogs) [7].

The major motivation for us for developing this method was the need to improve the characterization of MMGdi signals with the presence of cardiac interference. This is important because the study of MMGdi signals could be useful in clinical practice as an alternative non-invasive technique to evaluate respiratory muscle effort and to detect and quantify the severity of respiratory muscle weakness. In the current study we have only examined the SampEn at a single time scale. Costa et al. [19] developed a method that considers SampEn computed at several time-scales: multiscale entropy analysis. This method has been shown beneficial at differentiating between different cardiac diseases [19] and has allowed to examine the affect of fatigue and contraction intensity on the short and long-term complexity of biceps brachii surface electromyography [20]. Such approach can be useful for further analysis of the respiratory muscle effort by means of MMGdi signals.

In conclusion, we propose an algorithm for improving the evaluation of respiratory muscle effort from MMGdi signals that is robust against cardiac vibration interference.

## References

[1] Cramer JT, Housh TJ, Johnson GO, Ebersole KT, Perry SR, et al (2000) Mechanomyographic amplitude and mean power output during maximal, concentric, isokinetic muscle actions. Muscle Nerve 23: 1826–1831. 11102905 10.1002/1097-4598(200012)23:12<1826::aid-mus5>3.0.co;2-7

[2] Madeleine P, Farina D, Merletti R, Arendt-Nielsen L (2002) Upper trapezius muscle mechanomyographic and electromyographic activity in humans during low force fatiguing and non-fatiguing contractions. Eur J Appl Physiol 87: 327–336. 12172870 10.1007/s00421-002-0655-8

[3] Orizio C (1993) Muscle sound: bases for the introduction of a mechanomyographic signal in muscle studies. Crit Rev Biomed Eng 21: 201–243. 8243092

[4] Torres A, Fiz JA, Jané R, Laciar E, Galdiz JB, et al (2008) Rényi Entropy and Lempel-Ziv Complexity of Mechanomyographic Recordings of Diaphragm Muscle as Indexes of Respiratory Effort. Conf Proc IEEE Eng Med Biol Soc 2112–15. 19163113 10.1109/IEMBS.2008.4649610

[5] Bellemare F, Poirier C (2005) Diaphragm responses to stimulation. Physiological basis of respiratory disease. McGill University Health Centre; Montreal, Quebec. pp. 755–767.

[6] Sarlabous L, Torres A, Fiz JA, Morera J, Jané R (2013) Index for estimation of muscle force from mechanomyography based on the Lempel-Ziv algorithm. J Electromyogr Kinesiol 23: 548–557. 23428331 10.1016/j.jelekin.2012.12.007

[7] Sarlabous L, Torres A, Fiz JA, Gea J, Martinez Llorens JM, et al (2010) Interpretation of the Approximate Entropy using Fixed Tolerance Values as a Measure of Amplitude Variations in Biomedical Signals. Conf Proc IEEE Eng Med Biol Soc 5967–5970. 21096950 10.1109/IEMBS.2010.5627570

[8] Zanetti JM, Salerno DM (1991) Seismocardiography: a technique for recording precordial acceleration. 4th Annual IEEE Symposium on Computer-Based Medical Systems 4–9.

[9] Salerno DM, Zanetti J (1990) Seismocardiography: A New Technique for Recording Cardiac Vibrations. Concept, Method, and Initial Observations. J Cardiovasc Technol 9: 111–118.

[10] Nguyen H, Zhang J, Nam YH (2012) Timing detection and seismocardiography waveform extraction. Conf Proc IEEE Eng Med Biol Soc 3553–3556. 23366694 10.1109/EMBC.2012.6346733

[11] Petitjean M, Bellemare F (1994) Phonomyogram of the diaphragm during unilateral and bilateral phrenic nerve stimulation and changes with fatigue. Muscle Nerve 17: 1201–1209. 7935528 10.1002/mus.880171011

[12] Tavakolian K, Khosrow-Khavar F, Kajbafzadeh B, Marzencki M, Blaber AP, et al (2013) Precordial acceleration signals improve the performance of diastolic timed vibrations. Med Eng Phys 1–8. 10.1016/j.medengphy.2012.12.001 23291107

[13] Castiglioni P, Faini A, Parati G, Di Rienzo M (2007) Wearable seismocardiography. Conf Proc IEEE Eng Med Biol Soc 3954–3957. 18002865 10.1109/IEMBS.2007.4353199

[14] Laporta D, Grassino A (1985) Assessment of transdiaphragmatic pressure in humans. J Appl Physiol 58: 1469–1476. 3158636 10.1152/jappl.1985.58.5.1469

[15] Richman JS, Moorman JR (2000) Physiological time-series analysis using approximate entropy and sample entropy. Am J Physiol Hear Circ Physiol 278: H2039–H2049. 10.1152/ajpheart.2000.278.6.H2039 10843903

[16] Lake DE, Richman JS, Griffin MP, Moorman JR (2002) Sample entropy analysis of neonatal heart rate variability. Am J Physiol Regul Integr Comp Physiol 283: R789–97. 12185014 10.1152/ajpregu.00069.2002

[17] Richman JS, Lake DE, Moorman JR (2004) Sample entropy. Methods Enzymol 384: 172–184. 15081687 10.1016/S0076-6879(04)84011-4

[18] Molina-Picó A, Cuesta-Frau D, Aboy M, Crespo C, Miró-Martínez P, et al (2011) Comparative study of approximate entropy and sample entropy robustness to spikes. Artif Intell Med 53: 97–106. 21835600 10.1016/j.artmed.2011.06.007

[19] Costa M, Goldberger A, Peng CK (2005) Multiscale entropy analysis of biological signals. Phys Rev E 71: 021906. 10.1103/PhysRevE.71.021906 15783351

[20] Cashaback JG, Cluff T, Potvin JR (2013) Muscle fatigue and contraction intensity modulates the complexity of surface electromyography. J Electromyogr Kinesiol 23: 78–83. 22959820 10.1016/j.jelekin.2012.08.004

[21] Pincus SM (1991) Approximate entropy as a measure of system complexity. Proc Nati Acad Sci 88: 2297–2301. 10.1073/pnas.88.6.2297 PMC51218 11607165

[22] Zhang X, Zhou P (2012) Sample entropy analysis of surface EMG for improved muscle activity onset detection against spurious background spikes. J Electromyogr Kinesiol 22: 901–907. 22800657 10.1016/j.jelekin.2012.06.005 PMC3514830

[23] Madeleine P, Cescon C, Farina D (2006) Spatial and force dependency of mechanomyographic signal features. J Neurosci Methods 158: 89–99. 16808977 10.1016/j.jneumeth.2006.05.018

[24] Kaczmarek P, Celichowski J, Kasinski A (2005) Experimentally verified model of mechanomyograms recorded during single motor unit contractions. J Electromyogr Kinesiol 15: 617–630. 16055349 10.1016/j.jelekin.2005.03.005

[25] Kaczmarek P, Celichowski J, Drzymała-Celichowska H, Kasiński A (2009) The image of motor units architecture in the mechanomyographic signal during the single motor unit contraction: in vivo and simulation study. J Electromyogr Kinesiol 19: 553–563. 18455438 10.1016/j.jelekin.2008.03.007

[26] Uchiyama T, Hashimoto E (2011) System identification of the mechanomyogram from single motor units during voluntary isometric contraction. Med Biol Eng Comput 49: 1035–1043. 21394651 10.1007/s11517-011-0752-0

[27] Orizio C, Solomonow M, Diemont B, Gobbo M (2008) Muscle-joint unit transfer function derived from torque and surface mechanomyogram in humans using different stimulation protocols. J Neurosci Methods 173: 59–66. 18585787 10.1016/j.jneumeth.2008.05.012

[28] Orizio C, Baratta RV, Zhou BH, Solomonow M, Veicsteinas A (1999) Force and surface mechanomyogram relationship in cat gastrocnemius. J Electromyogr Kinesiol 9: 131–140. 10098713 10.1016/s1050-6411(98)00044-3

[29] Uchiyama T, Sakai H (2013) System identification of evoked mechanomyogram from abductor pollicis brevis muscle in isometric contraction. Med Biol Eng Comput 51: 1349–1355. 23934080 10.1007/s11517-013-1107-9

[30] Tobin M, Mador M, Guenther S, Lodato R, Sackner M (1998) Variability of resting respiratory center drive and timing in healthy subjects. J Appl Physiol 65: 309–317. 10.1152/jappl.1988.65.1.309 3403474

[31] American Thoracic Society/European Respiratory Society (2002) ATS/ERS Statement on Respiratory Muscle Testing. Am J Respir Crit Care 166: 518–624. 10.1164/rccm.166.4.518 12186831

[32] Sinderby C, Lindström L, Grassino AE (1985) Automatic Asessment of Electromyogram Quality. J Appl Physiol 79: 1803–15. 10.1152/jappl.1995.79.5.1803 8594044

[33] Sarlabous L, Torres C, Fiz JA, Morera J, Jané R (2012) Evaluation and adaptive attenuation of the cardiac vibration interference in mechanomyographic signals. Conf Proc IEEE Eng Med Biol Soc 3400–3403. 23366656 10.1109/EMBC.2012.6346695

[34] Sarlabous L, Torres A, Fiz JA, Jané R (2013) Cardiac Interference Reduction in Diaphragmatic MMG Signals during a Maintained Inspiratory Pressure Test. Conf Proc IEEE Eng Med Biol Soc 3845–3848. 24110570 10.1109/EMBC.2013.6610383

